# Chemoprophylaxis in addition to mechanical prophylaxis after total knee arthroplasty surgery does not reduce the incidence of venous thromboembolism

**DOI:** 10.1186/s12959-019-0200-1

**Published:** 2019-06-20

**Authors:** Jing Loong Moses Loh, Stephrene Chan, Keng Lin Wong, Sanjay de Mel, Eng Soo Yap

**Affiliations:** 1 Department of Orthopaedic Surgery, Singapore General Hospital, 20 College Road, Academia, Level 4, Singapore, 169865 Singapore; 2 Department of Haematology, Tan Tock Seng Hospital, Singapore, Singapore; 3 Department of Orthopaedic Surgery, Sengkang General Hospital, Singapore, Singapore; 4 Department of Hematology-Oncology, National University Cancer Institute, National University Hospital, Singapore, Singapore

## Abstract

**Background:**

Venous thromboembolism (VTE) of the lower limbs is an important complication post total knee arthroplasty (TKA). Current guidelines recommend routine chemical prophylaxis to all patients undergoing this procedure but this is rarely done in Asia as it is believed that Asians have a lower risk of VTE. However, recent evidence suggests otherwise.

**Aims:**

We evaluated the incidence of DVT after TKA in a multi-ethnic Asian population with and without pharmacological prophylaxis, as well as the management and outcome of patients with post-operative DVTs.

**Methods:**

We conducted a retrospective study of consecutive patients who underwent TKA in our hospital from 1st January 2004 to 30th December 2014. All patients were on mechanical thromboprophylaxis via calf pumps after TKA with a postoperative day 3 to 5 doppler ultrasound (DUS) of bilateral lower limbs. 2258 (80.7%) patients did not receive additional chemoprophylaxis, while 540 (19.3%) received chemoprophylaxis on top of mechanical thromboprophylaxis. All patients who received chemoprophylaxis were administered the drug until they were ambulating, with a median administration duration of 6 days. Patients were followed up for a period of 3 months for recurrence of DVTs and 24 months for postoperative outcome scores.

**Results:**

Two thousand nine hundred seventy-eight patients had DUS of the lower limbs with 134 diagnosed with DVT giving an incidence of 4.5%. Six of these patients had concurrent PEs. There were 26 (19.4%) proximal DVTs and 108 (80.6%) distal DVTs. After 3 months of follow up, no additional VTE occurred. None of the DVTs or PEs progressed.

All DVTs with accompanying PE were proximal. 102 out of 2200 patients (4.6%) without chemoprophylaxis developed DVT as compared to 32 out of 540 patients (5.9%) with chemoprophylaxis, which was not statistically significant (*p* = 0.13). 19 (0.8%) proximal and 83 (3.8%) distal DVT developed in the patient group without chemoprophylaxis while 4 (0.7%) proximal and 28 (5.2%) distal DVT developed in the patient group with (*p* = 0.62). Comparison of the incidence of PEs between the two groups, revealed a similar incidence with 5 out of 2200 patients (0.2%) without chemoprophylaxis developing PE as compared to 1 out of 540 patients (0.2%) with chemoprophylaxis (*p* = 0.87).

In addition, patients with chemoprophylaxis showed an association with higher post-operative outcome scores such as post op 6 months SF36 (PCS), post op 12 months SF36 (PCS), post op 12 months SF36 (MCS), post op 24 months SF36 (MCS) and post op 24 months WOMAC.

**Conclusion:**

In one of the largest Asian studies specifically investigating the incidence of DVT after TKA, we found that the incidence is low at 4.5%. This is in contrast to recent studies that showed higher post-operative VTE rates similar to Western populations. In addition, patients who were administered chemoprophylaxis did not have a statistically significant difference in incidence of VTE although it did show a correlation with higher post-operative outcome scores which may indicate better function. This was seen in functional outcome scores such as post op 6 months SF36 (PCS), post op 12 months SF36 (PCS), post op 12 months SF36 (MCS), post op 24 months SF36 (MCS) and post op 24 months WOMAC.

## Introduction

There are over 1 million total hip and total knee replacement procedures performed each year in the United States alone [1]. Demand for hip and knee replacements is rising annually, and growth is expected to be substantial in the years to come due to various reasons including higher rates of diagnosis and treatment of advanced arthritis, as well as increasing demand for improved mobility and quality of life [1]. Venous thromboembolic events (VTE), including deep vein thrombosis (DVT) and pulmonary embolism (PE), are amongst one of the leading causes of morbidity and mortality associated with total knee arthroplasty (TKA) surgeries. 40–50% of patients with untreated symptomatic DVT will develop a PE within 3 months and 10% of patients with symptomatic PE die within an hour of onset [2]. In Caucasians, the DVT incidence following total knee arthroplasty (TKA) is reported at 41–85% and the incidence of pulmonary embolism (PE) of 1.5–10% [3]. The incidences of DVT reported in Asia following various orthopaedic procedures vary and have been reported to be as high as 53.3% [4–6]. In our local context, the reported rate of DVT after TKA is 14% [7].

The efficacy of chemical and mechanical thromboprophylaxis in preventing VTEs in this high-risk group of patients has been well demonstrated. The American Academy of Orthopaedic Surgeons (AAOS) recommends the use of pharmacologic agents and/or mechanical compressive devices for the prevention of venous thromboembolism in patients undergoing elective hip or knee arthroplasty, and who are not at elevated risk beyond that of the surgery itself for venous thromboembolism or bleeding [8]. Similarly, the American College of Chest Physicians (ACCP) states that in patients undergoing TKA, post-surgical chemical thromboprophylaxis with low-molecular-weight heparin (LMWH) or factor Xa inhibitors is recommended for a minimum of 10 to 14 days to reduce the risk and incidence of symptomatic deep vein thrombosis (DVT) and pulmonary embolism (PE) [9].

Despite this, chemoprophylaxis is generally under-practiced in the Asian context [10], owing to concerns of bleeding, slow wound healing and prolonged wound drainage [11, 12]. In a recent paper, the bleeding from LMWH (enoxaparin) use after TKA in an Asian population was shown to be as high as 20% [13]. This reluctance to practise postoperative chemoprophylaxis is reinforced by the belief that the incidence of VTEs has been reported to be lower in the Asian population as compared to the Caucasian population [10]. This raises a debate about whether routine postoperative chemoprophylaxis is required in Asian patients [14].

The primary aim of this study is to determine the incidence of postoperative VTE following TKA in our local population with and without additional chemoprophylaxis in addition to routine mechanical prophylaxis in all patients. The secondary aims are to assess possible risk factors associated with VTE and to compare the 2 year functional outcomes of patients with and without postoperative VTE. We hypothesize that the rate of VTE in TKA patients is comparable to that of Western populations, and that there would be no differences in terms of functional outcomes between patients with and without postoperative VTE.

## Materials and methods

We conducted a retrospective study of all patients who underwent elective TKA from 1st January 2004 to 31st December 2014 at an academic tertiary hospital for osteoarthritis of the knee. Ethics approval was obtained from the Institutional Review Board. Electronic and paper records were reviewed. The data was collected from case notes review, anaesthetic assessment chart reviews and electronic notes. Inclusion criteria were all patients who had underwent elective TKA during the specific time period. Patients who had total knee arthroplasty due to fractures, had unicompartmental knee arthroplasty and who were pre-operatively non ambulatory or minimally ambulatory, were excluded from the study.

### Post-operative mobilisation and thromboprophylaxis protocol

Patients were allowed to stand on the first postoperative day and progressed to full-weight bearing activity with walking aids as tolerated. Each patient was provided with mechanical prophylaxis immediately post operation, which involved the use of both intermittent pneumatic compression pumps (ArjoHuntleigh Flowtron® Excel DVT pump system) and thromboembolic deterrent open toe knee length compression stockings (T.E.D™ Knee Length Anti- Embolism Stockings). Additional chemical thromboprophylaxis was administered, subject to the preference of the surgeon and the patient’s risk profile. Mechanical prophylaxis was continued until patients were able to ambulate confidently with walking aids for two physiotherapy sessions on the same day. All patients underwent a DUS of both lower limbs within five days after their operation to detect DVT as part of a hospital wide protocol.

### Data collection

Patient demographics including age, gender, significant co-morbidities, and pre-operative mobility status were recorded. Thrombotic risk factors such as hypertension, ischemic heart disease, Diabetes Mellitus, smoking habits, congestive heart failure, previous lower limb VTE and cancer were assessed.

The absence or presence of chemoprophylaxis use, in the form of subcutaneous low molecular weight heparin, was recorded for these patients, together with the presence or absence of VTE. The use of other anti-thrombotic agents postoperatively such as anti-platelet therapy was recorded The presence of DVT was defined as the lack of compressibility and impedance of normal blood in the affected veins, as seen on DUS, with the trifurcation point of the popliteal vein used as the demarcation between proximal and distal deep vein thrombosis. Patients who had symptoms suggestive of PE underwent computed tomography pulmonary angiogram (CTPA) for confirmation of PE, and the presence of symptomatic PE was also documented. Patients were followed up for three months to monitor for any recurrence of VTE.

All patients were followed up with outcome scores for 2 years post operation. Western Ontario and McMaster Universities Osteoarthritis Index [WOMAC] pain), and Short Form-36 (SF-36). Patient outcomes were recorded at six months, twelve months and twenty four months after surgery. These scores enable physicans to have an objective method of assessing the patient’s function pre operation and postoperation, and can also be used to assess the patient’s general wellbeing.. The WOMAC consists of 24 items divided into 3 subscales – Pain (WOMAC 1), Stiffness (WOMAC 2) and Physical Function (WOMAC 3). The 3 WOMAC subscales were normalized to 0–100 scales to correct for differences in scale range by using the methods recommended by Bellamy. The higher the score, the better the outcomes [15]. With regards to SF-36, the SF-36 is a 36-item patient-reported survey of patient health and reflects quality of life. It consists of eight scaled scores, which are the weighted sums of the questions in their section. Each scale was transformed into a 0–100 scale on the assumption that each question carries equal weight. The higher the score, the less disability; a score of zero is equivalent to maximum disability and a score of 100 is equivalent to no disability. The eight sections are: vitality (SFVI), physical functioning (SFPF), bodily pain (SFBP), general health perceptions (SFGHP), physical role functioning (SFPRF), emotional role functioning (SFERF), social role functioning (SFSF) and mental health (SFMH) [16].

Patients who underwent therapeutic anti-coagulation therapy for DVT and/or PE were followed up for a minimum of 3 months to document any bleeding complications from anti-coagulation therapy.

### Statistical analysis

Univariate analyses were performed to describe the presence of DVT in relation to the demographic variables, comorbidities and surgical factors. Mann-Whitney U test was used for numerical prognostic factors, and chi-square test was used for categorical factors. As we noted differences in the age and sex of the patients, and the presence of cancer and previous VTE have been previously shown to demonstrate an increased risk in developing VTE, we adjusted our odds ratios for these four factors.

This was followed by multivariate logistic regression, performed using the variables identified as significant from the univariate analysis. A two-sided *p*-value of < 0.05 was used to select for these variables to be included in in multivariate logistic regression. Subsequent analysis also took a *p*-value of < 0.05 as unlikely to be due to chance. The logistic regression method used was Forward: Conditional, with an entry probability of 0.05 and a removal probability of 0.10.

Univariate analysis was also done to describe the type of prophylaxis in relation to functional outcome scores such as the post-op 6 month scores, post-op 12 months scores and post op 24 months scores.

All analysis was performed using IBM Statistical Package for Social Sciences (SPSS) Version 22.

## Results

Using the above-mentioned criteria, 2978 patients were included in this 10-year study (2009 Chinese, 360 Malays, 390 Indians, 35 others). There were 725 men and 2073 women, with a mean age of 65.86. Mean body mass index was 27.85 kg/m2 (range, 24 to 31.5 kg/m2). This is illustrated in Table 1. All patients received mechanical thromboprophylaxis.

**Table 1:** Univariate analysis of Demographics, Comorbidities and Surgical Factors in relation to presence of DVT after TKR

Characteristic		Presence of DVT		Odds Ratio (95% Confidence Interval)	*P*-Value	Adjusted Odds Ratio (95% Confidence Interval)	*P*-value
	Total	Yes	No				
Demographic Variables							
Sex				1.77(0.91–1.86)	0.184		
Male	725	41 (5.7)	684 (94.3)				
Female	2073	150 (7.1)	1926 (92.9)				
Age (years)					0.671		
Median (range)		66.06 (40)	65.78 (58)				
Minimum and maximum	31 and 90						
Ethnicity				7.81 (7.21–8.456)	0.05	1.095 (0.89–1.349)	0.39
Chinese	2009 (72.7)	137 (6.8)	1872 (93.2)				
Malay	360 (11.8)	18 (5.0)	342 (95.09)				
Indian	394 (14.5)	27 (6.9)	367 (93.1)				
Others	35 (1.0)	6 (82.9)	29 (17.1)				
BMI				2.003 (1.783–2.412)	0.55	1.003 (0.973–1.035)	0.84
Median (range)		27.95 (28.63)	27.71 (52.46)				
Interquartile Range		5.45	5.92				
Comorbidities							
Hypertension				1.1.1.1.1 0.70 (0.60–1.09)	0.13	0.859 (0.602–1.224)	0.40
Y		111 (7.0)	1392 (93.0)				
N		80 (6.2)	1218 (93.8)				
Ischemic Heart Disease				1.1.1.1.1 1.00 (0.62–1.66)	0.99	1.000 (0.549–1.837)	0.99
Y		19 (6.7)	263 (93.3)				
N		172 (6.8)	2347 (93.2)				
Diabetes Mellitus				1.1.1.1.1 1.00 (0.69–1.48)	0.96	1.215 (0.78–1.893)	0.388
Y		34 (6.8)	468 (93.2)				
N		157 (5.8)	2539 (94.2)				
Cancer				0.65 (0.33–1.62)	0.42		
Y		7 (8.9)	71 (91.1)				
N		184 (8.3)	2036 (91.7)				
Smoking				0.06 (0.29–5.01)	0.81	1.29 (0.261–6.377)	0.755
Y		2 (5.7)	33 (94.3)				
N		189 (6.8)	2577 (93.2)				
Consumption of Anti-Platelet Drugs				0.56 (0.47–1.45)	0.45	0.834 (0.400–1.738)	0.628
Y		14 (8.1)	159 (91.9)				
N		177 (6.7)	2451 (93.3)				
Consumption of NSAIDs				0.88 (0.72–2.00)	0.54	1.297 (0.697–2.415)	0.412
Y		17 (5.7)	273 (94.3)				
N		174 (6.9)	2337 (93.1)				
Previous Lower Limb VTE				9.85 (1.23–10.98)	0.00		
Y		5 (24.0)	16 (76.0)				
N		187 (6.7)	2592 (93.3)				
Family History of VTE				1.1.1.1.1 1.41 (0.02–1.66)	0.24	3.777 (0.063–22.518)	0.525
Y		1 (25.0)	4 (75.0)				
N		190 (6.8)	2606 (93.2)				
Surgical Factors							
Length of Stay		Y	N			1.152 (1.110–1.194)	0.000
Median (range)		9.33 (60)	6.42 (28)				
Interquartile Range		5	2				
Operation Time (minutes)				2.000 (0.954–3.025)	0.55	1.000 (0.996–1.005)	0.889
Median (range)		121 (213)	119 (353)				
Interquartile Range		43	28				

Two thousand two hundred fifty-eight (80.7%) patients did not receive additional chemoprophylaxis, while the remaining 540 (19.3%) received chemoprophylaxis along with mechanical thromboprophylaxis. All patients who received chemoprophylaxis were administered the enoxaparin until they were ambulating, with a median administration duration of 6 days (minimum 2 days, maximum 30 days).

### Overall rates of VTEs

Two thousand nine hundred seventy-eight patients had DUS of the lower limbs with 134 diagnosed with DVT with an incidence of 4.5%. There were 26 (19.4%) proximal DVTs and 108 (80.6%) distal DVTs. In addition, there were 6 PEs diagnosed on computer tomography of the chest. At 3 months of follow up, no additional VTE occurred.

Table 2 shows the sites of thrombi identified by the duplex Doppler ultrasound. All DVTs with accompanying PE were proximal.

**Table 2:** Sites of Lower Limb Venous Thromboses by Number and Percentage

Site	Number of Patients
Knees with no thrombi	2978
Knees with DVT	134 (100%)
Proximal DVT	26 (19.4%)
Superficial Femoral Vein	6
Popliteal Vein	20
Distal DVT	108 (80.6%)
Soleal	14
Peroneal	64
Posterior Tibial	30
Superfical Venous Thromboses	
Calf (Intramuscular)	27
Calf (Superficial)	2
Great Saphenous	15

Table 3 shows the method of treatment for the identified DVTs. Almost 50% were simply monitored while the other 50% were anticoagulated. Treatment of the DVTs was heterogeneous - in some cases, haematologists were consulted while in some cases, they were not. All proximal DVTs were anticoagulated. Out of the distal DVTs, only posterior tibial veins were not anticoagulated.

**Table 3:** Type of Treatment for Patients with Lower Limb Venous Thromboses

Type of DVT Treatment	Number of Patients
Monitor	66
Anticoagulation	67

#### Subgroup analysis: comparison between patients with and without chemoprophylaxis

One hundred two out of 2200 patients (4.6%) without chemoprophylaxis developed DVT as compared to 32 out of 540 patients (5.9%) with chemoprophylaxis, which was not statistically significant (*p* = 0.13). 19 (0.8%) proximal and 83 (3.8%) distal DVT developed in the patient group without chemoprophylaxis while 4 (0.7%) proximal and 28 (5.2%) distal DVT developed in the patient group with (*p* = 0.62). Comparison of the incidence of PEs between the two groups, revealed a similar incidence with 5 out of 2200 patients (0.2%) without chemoprophylaxis developing PE as compared to 1 out of 540 patients (0.2%) with chemoprophylaxis (*p* = 0.87). 2 patients had bleeding complications – one from the group with chemoprophylaxis and one from the group without chemoprophylaxis.

Table 4 shows the comparison of the functional outcome scores with and without chemical prophylaxis. Post op 6 months SF36 (MCS), post op 12 months SF36 (MCS), post op24 months SF36 (MCS), and post op 24 months WOMAC are statistically significant.

**Table 4:** Functional Outcome Scores of Patients with and without Chemoprophylaxis

Type of Outcome	With chemical prophylaxis	Without chemical prophylaxis	*P*-value
Post-Op 6 months SF-36 PCS	85.6	82.5	0.682
Post-Op 6 months SF-36 MCS	78.6	94.3	0.00
Post-Op 6 months WOMAC	82.6	83.0	0.914
Post-Op 12 months SF-36 PCS	76.0	74.4	0.118
Post-Op 12 months SF-36 MCS	82.4	68.8	0.00
Post-Op 12 months WOMAC	70.8	72.3	0.567
Post-Op 24 months SF-36 PCS	55.3	55.8	0.573
Post-Op 24 months SF-36 MCS	61.7	54.6	0.006
Post-Op 24 months WOMAC	51.9	56.5	0.08

Table 5 shows the results of multivariate analysis using demographics, comorbidities, and surgical factors found to be significant on univariate analysis. For multivariate analysis, length of stay and previous lower limb VTE are significant.

**Table 5:** Multivariate analysis using demographics, comorbidities and surgical factors found to be significant on univariate analysis

Statement	Odds Ratio (95% Confidence interval)	*P*-value
Ethnicity		0.089
Chinese	0.184	
Malay	0.400	
Indian	0.275	
Others	0.354	
Length of Stay	1.134 (1.101–1.168)	0.00
Previous Lower Limb VTE	3.963 (1.327–11.831)	0.014

Table 6 shows the results of multivariate analysis using the postoperative outcome scores which were found to be significant on univariate analysis. For multivariate analysis, post op 6 months SF36 (PCS) and post op 24 months SF36 (MCS) are significant. The analysis showed that the type of thromboprophylaxis was independently associated with post op 6 months SF36 (PCS), post op and post op 24 months SF36 (MCS).

**Table 6:** Multivariate Analysis Using Postoperative Outcome Scores Found to be Significant on Univariate Analysis

Statement	Odds Ratio (95% Confidence interval)	*p*-value
post op 6 months SF36 (MCS)	1.182 (0.566–1.571)	0.032
post op 12 months SF36 (MCS)	0.971 (0.569–1.574)	0.132
post op 24 months SF36 (MCS)	1.063 (0.573–1.578)	0.014
post op 12 months WOMAC	1.342 (0.888–1.900)	0.21

## Discussion

We showed that the incidence of DVTs in a multi-ethnic Asian population post elective total knee arthroplasty with mechanical prophylaxis is 4.6%. The use of mechanical and chemical prophylaxis did not lower the risk of developing DVT (incidence of 5.9%). Comparisons between the patient groups with and without chemoprophylaxis revealed that all DVT that developed with the use of chemoprophylaxis were proximal DVTs, suggesting that chemoprophylaxis could possibly have reduced the incidence of distal DVT more effectively than that of proximal DVT. This observation could also possibly explicate the relative ineffectiveness of chemoprophylaxis in reducing the incidence of pulmonary embolism, since pulmonary emboli were more strongly correlated with proximal DVT as found in our study. This observation has been noted in a few previous studies of total knee arthroplasty, where chemoprophylaxis has been found to be ineffective in reducing the incidence of PE [17, 18].

Patients who undergo total knee arthroplasty have been identified to have a high risk for VTE, leading to recommendations in international guidelines to use a combination of mechanical and chemical thromboprophylaxis before and after the procedure [19, 20].

Pharmacological thromboprophylaxis has been recommended to reduce the prevalence of postoperative DVT on the assumption that it will reduce the prevalence of PE, mortality, and thrombophlebitic syndrome [21, 22]. However, pharmacological thromboprophylaxis itself carries several risks such as the risk of bleeding, complications of blood loss, transfusion, transfusion-related transmission of disease, wound-healing problems, hematoma, slowed rehabilitation, wound drainage, and infection [23]. The practice of routine postoperative chemical thromboprophylaxis to prevent VTE has mainly been based on Western literature thus far, and the recent acknowledgement of studies which show a lower incidence of thromboembolism in Asia [24] has led to questions regarding the need for routine chemoprophylaxis for patients undergoing total knee arthroplasty.

Recent studies done in Asian populations however have shown otherwise, with the incidence of DVT being shown to be comparable in both Caucasian and Asian populations [25–27]. Treatment should ideally be individualized to the patient’s risk profile, raising the question of Asian populations possibly following different guidelines for thromboprophylaxis after TKA. Current guidelines recommend differing modalities of thromboprophylaxis depending on the patient’s risk factors. The AAOS recommends that patients who have had a previous VTE should receive the combined modalities of pharmacologic prophylaxis and mechanical prophylaxis, such as thromboembolic deterrent (TED) stockings or intermittent pneumatic compressive devices (IPCD). In patients who have a known bleeding disorder (e.g., hemophilia) and/or active liver disease, only mechanical compressive devices should be used for preventing VTE [8]. This is identical to the ACCP which also advocates combined modalities for normal patients and solely mechanical prophylaxis or no prophylaxis for patients with an increased risk of bleeding [9]. Significantly, in a multicenter randomized controlled trial comparing IPCD against enoxaparin, Colwell et al. showed that IPCD was just as effective as enoxaparin in preventing proximal and distal DVT and PE events in hip arthroplasty patients, but resulted in a much lower bleeding risk (1.3% IPCD vs 4.3% LMWH) [28]. This adds to the argument that patients with a decreased risk of VTE should not undergo chemoprophylaxis but use only mechanical prophylaxis, thereby sparing them the adverse risk of bleeding. However, it is still unclear whether combined modalities of chemical and mechanical prophylaxis are better than either chemoprophylaxis or mechanical prophylaxis alone in overall functional outcomes. A study in 2013 found that there were no significant differences in the patient-reported quality of life outcomes and therapist-reported knee range of motion between patients who had developed DVT and those who had not [17]. Our study showed that the type of thromboprophylaxis was independently associated with post op 6 months SF36 (PCS) and post op 24 months SF36 (MCS). Anticoagulation has been associated with decreased risk of acute myocardial infarction and stroke [29]. Given our patient demographics, it is possible this could have played a role in higher patient outcome scores despite no significant difference in VTE incidence. Further studies will have to be done to explore this finding.

Our incidence of VTE is low compared to that of Caucasian populations. In a review of the literature, there is only a single study on a Caucasian population which revealed similar a similar incidence of DVT -Gelfer et al. showed an incidence of DVT of 6.6% in a THA and TKA population that was treated only by mechanical prophylaxis therapy coupled with aspirin [30]. An explanation for this difference between the Asian and Western populations is most likely due to the prevalence of prothrombotic factors in Caucasians which cause them to have a higher risk of developing VTEs as compared to Asians, regardless of the type of surgery performed [31]. In fact, the low probability of Asian populations having postoperative DVT and PE as compared to Western populations was first highlighted by Tinckler et al. in 1964 [32]. There are some data that support genetic differences as a partial cause of a lower risk of VTE in Asians. The most common of the known genetic mutations is a gene known as factor V Leiden [33] which increases DVT risk by about 7 times in heterozygotes, and about 80 times in homozygotes. It has been shown to be found in approximately 5% of Caucasians, but is less common in Africans and rare in Asians [34]. Another genetic trait that predisposes to DVT and PE is the prothrombin promoter G20210A mutation. This is found in 4 to 6% of Caucasians and enhances transcription of the prothrombin gene, yielding higher prothrombin levels and consequently easier generation of thrombin, the key enzyme in blood clotting [35].

Last but not least, our study noted an apparent correlation between proximal DVT as well as bilateral DVT and the development of PE. All the PEs that had an accompanying DVT in the study had proximal DVT. Li et al. had identified a similar relationship in her study which showed the risk of developing a silent PE in patients with proximal DVT [36]. Larger scale studies would be beneficial in further investigating this possible relationship. Strengths of our study are that we have a large cohort of patients in a 10 year follow up period with no loss to follow up in the public sector. This is due to our National Electronic Healthcare Registry in Singapore which combines patient information in all our public hospitals. However as it does not include the private hospitals, there could have been loss to follow up to private institutions during the study period. Care was also standardized across the study as the methods were part of the hospital protocol. It is also one of the first studies to show a difference in functional outcomes scores (SF-36) between the type of thromboprophylaxis for total knee arthroplasty. A limitation of our study is that we employed the usage of Doppler Ultrasound, which is operator dependent. Thus we could have missed out on certain DVTs that could have been picked up by venograms.

In Asian patients on thromboprophylaxis post-TKA, there is no significant difference in incidence of DVT between patients on chemoprophylaxis to those without. Mechanical postoperative thromboprophylaxis may be adequate in post-TKA DVT prevention, in our local context. A randomized controlled trial in Asians should be done to assess if the addition of chemoprophylaxis on top of mechanical thromboprophylaxis might lead to more harm than good.

## Conclusion

In one of the largest Asian studies specifically investigating the incidence of DVT after TKA, we found that the incidence is low at 4.5%. The study institution’s policy for VTE prevention included routine mechanical thromboprophylaxis and duplex ultrasound. With this policy, VTE rates that are clinically relevant were very low.

This is in contrast to recent studies that showed higher post-operative VTE rates similar to Western populations.. In addition, patients who were administered chemoprophylaxis did not have a statistically significant difference in incidence of VTE although it did show a correlation with higher post-operative outcome scores indicating better function.

## Abbreviations

BMI: Body Mass Index
CI: Confidence Interval
DVT: Deep Venous Thrombosis
OR: Odd Ratio
PE: Pulmonary Embolism
SF-36: Short Form-36
TKA: Total Knee Arthroplasty
VTE: Venous Thromboembolism
WOMAC: Western Ontario and McMaster Universities Osteoarthritis Index
